# 3D Printed Microfluidic Devices for Drug Release Assays

**DOI:** 10.3390/pharmaceutics13010013

**Published:** 2020-12-23

**Authors:** Benzion Amoyav, Yoel Goldstein, Eliana Steinberg, Ofra Benny

**Affiliations:** The Institute for Drug Research, School of Pharmacy, The Faculty of Medicine, The Hebrew University of Jerusalem, Jerusalem 91120, Israel; benzion.amoyav@mail.huji.ac.il (B.A.); yoel.goldstein@mail.huji.ac.il (Y.G.); eliana.steinberg@mail.huji.ac.il (E.S.)

**Keywords:** microfluidics, 3D printing, chip manufacturing, microfabrication, dissolution test, microspheres, porous

## Abstract

Microfluidics research for various applications, including drug delivery, cell-based assays and biomedical research has grown exponentially. Despite this technology’s enormous potential, drawbacks include the need for multistep fabrication, typically with lithography. We present a one-step fabrication process of a microfluidic chip for drug dissolution assays based on a 3D printing technology. Doxorubicin porous and non-porous microspheres, with a mean diameter of 250µm, were fabricated using a conventional “batch” or microfluidic method, based on an optimized solid-in-oil-in-water protocol. Microspheres fabricated with microfluidics system exhibited higher encapsulation efficiency and drug content as compared with batch formulations. We determined drug release profiles of microspheres in varying pH conditions using two distinct dissolution devices that differed in their mechanical barrier structures. The release profile of the “V” shape barrier was similar to that of the dialysis sac test and differed from the “basket” barrier design. Importantly, a cytotoxicity test confirmed biocompatibility of the printed resin. Finally, the chip exhibited high durability and stability, enabling multiple recycling sessions. We show how the combination of microfluidics and 3D printing can reduce costs and time, providing an efficient platform for particle production while offering a feasible cost-effective alternative to clean-room facility polydimethylsiloxane-based chip microfabrication.

## 1. Introduction

In recent decades, microfluidic research has gained tremendous momentum and opened a new era for advanced and innovative research down to the micron and submicron scale. Similar to cutting-edge sensors and pump platforms, these micron-scale chips can gently manipulate fluid flow with small sample volumes, thus providing alternative and more efficient systems in a broad range of applications (e.g., biology, chemistry, physics, engineering and pharmaceutics). These are characterized by high-throughput and parallel analysis leading to overall reduced costs [1,2,3]. A common platform for microparticle formation using the microfluidic approach is the droplet-based flow focusing chip, in which two or more liquid phases intersect to form monodisperse particles in a controlled manner [4]. Generally, microparticles and nanoparticles fabricated with the microfluidic approach are characterized by a low polydispersity index, improvement in encapsulation efficiency (EE) and drug content (DC) rates, which are comparable to those in conventional synthesis methods [5,6].

Additive manufacturing (AD), or three-dimensional (3D) printing, is a growing alternative technology for rapid prototyping of simple and complex structures for various applications [7]. Among AD methods, digital light processing (DLP) has emerged as an attractive 3D printing technology, due to both its high printing resolution and its relatively low costs. This has led to its widespread availability to private consumers and research institutions. In this technique, the printed objects are built from a resin in a layer-by-layer configuration by controlled UV photopolymerization, thus providing a simple alternative to conventional fabrication methods [8].

Until recently, soft lithography was the most common method for fabricating microfluidic-based poly-dimethyl-siloxane (PDMS) devices for biomedical and research applications. This was mainly due to the low cost of PDMS, its biocompatibility and its high transparency [9]. However, fabrication of microfluidic chips using this method requires clean-room facilities and special training. Moreover, this process can be costly and time-consuming due to the several tedious steps required, the low availability of the necessary advanced equipment, rectilinear feature restriction and the multiple attempts that may be needed to achieve the final product. Another downside of using PDMS-based devices for various microfluidic applications is that it sometimes can absorb small molecules (e.g., hydrophobic drugs), thus distorting the results. Furthermore, the fabricated chips are not recyclable, tend to dry or tear after a short time and are generally characterized by low durability [10,11].

Microparticle and nanoparticle-based drug delivery systems (DDSs) are considered a promising approach for targeted therapy in various diseases [12,13,14]. These drug carriers can be designed to overcome many of the limitations that a conventional DDS faces, such as low bioavailability, drug stability and solubility, slow release, adverse side effects and systemic toxicity. Consequently, microparticle and nanoparticle-based DDS may provide a safer and more effective tool for selective delivery treatment [15]. A fundamental and essential component of the research and development of novel DDS are the analytical tests required, including formulation stability, chemo-physical characterization, and shelf-life. One of the most basic and necessary tests used for novel solid DDS is the dissolution test, which determines the drug release kinetics profile. This test measures the rate and extent of release from a dosage form and monitors changes in formulation over a predetermined time course [16]. In the early development stage, the dissolution test aids in the formulation optimization properties (e.g., in-vitro–in-vivo correlations). In later development phases, the test ensures consistency between production batches and serves as a tool for the approval of generic products. Dissolution tests are important for verifying therapeutic effectiveness and for creating a streamlined process within quality control and for standardized manufacturing [17].

The initial steps in formulation development require many calibrations until a final dosage form is defined. This is due to the polymer or matrix composition, the molecular weight of the matrix, and the physicochemical properties of the embedded drugs. Any change in the formation parameters may affect release kinetics from the dosage form, thus necessitating proper characterization. Current practice relies mainly on large and expensive dissolution apparatus, which are typically defined in the pharmacopeia (e.g., Apparatus 1 (basket) and Apparatus 2 (paddle), Apparatus 3 (reciprocating cylinder), Apparatus 4 (flow-through cell) or by varying types of dialysis methods for in-vitro release studies [18,19,20]. However, most of the apparatus devices show high variability that may yield different release profiles for the same tested product [21,22].

Standard dissolution devices are generally designed for large dimensions and require a high quantity of test material, whereas many of the novel advanced DDS are based—especially in the development phase—on the production of small amounts of microparticles or nanoparticles. Moreover, proper operation of standard dissolution apparatus requires special training, and due to the high costs, they are mostly available to industry [23]. Dialysis methods are currently the most popular technique for drug release measurements in laboratory practices. According to this method, a drug is released from the DDS and diffuses through a dialysis wall membrane to the outer compartment, where it is sampled for further analysis. Despite its simplicity, this method has major disadvantages. If the dialysis bag is incorrectly sealed, leakage of media and DDS may occur. Wrong choice of a molecular cut-off might lead to a drug-transport limiting factor instead of rapid diffusion to the outer compartment. Furthermore, in this method, sink conditions might not be reached and drugs or DDS might chemically or electrostatically bind to the dialyzing membrane. This would lead to incomplete or misleading drug release data [24,25]. The above-mentioned limitations emphasize the need for a novel, cost-effective and simple, reliable method of drug release that is adjusted to small volume formulations.

**Figure 1:**
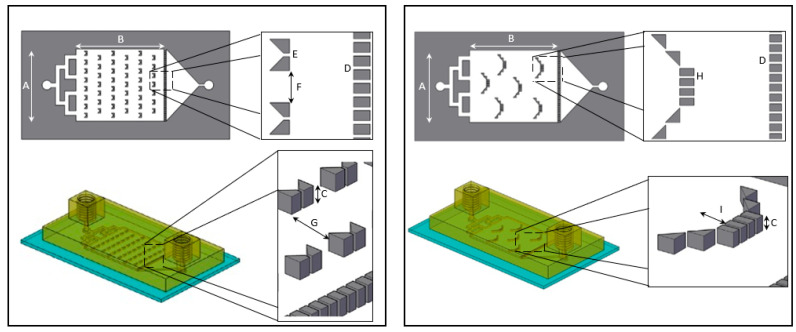
Overhead and side view schematic illustration of “V” (left) and “basket” (right) shape dissolution microfluidic chip. The overall dimensions of the “V” and “basket” shape chips are identical with a main chamber width (**A**) of 16 mm, length (**B**) of 20 mm and height (**C**) of 600 µm. Both chips contain a downstream trap of 38 teeth separated by 120 µm gaps (**D**). “V” trap design (left): includes two trapezoids separated by 120 µm gap (**E**). The V traps are lined in six rows of on and off seven and eight traps, with a 1 mm gap between traps (**F**) and separated by 2.4 mm gap (**G**). “Basket” trap design (right): includes four rectangular teeth and additional two diagonal triangular teeth placed on both sides, separated by 120 µm gaps (**H**) creating a 1.2 mm depth (**I**).

Here we present a “plug and play”, a recyclable and durable microfluidic drug release chip, that is highly reproducible and easy to use. We designed two distinct chip geometries, “basket” and “V” shape barriers (Figure 1), previously used for cell membrane receptor screening [26], and demonstrated drug release assays from both.

**Figure 2:**
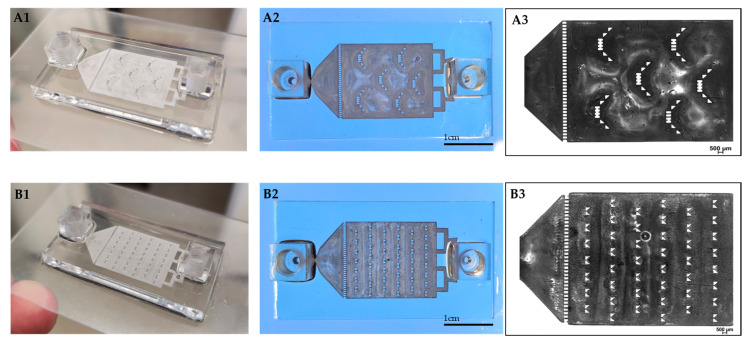
3D printed drug-release chips containing different interior particle trap designs. “Basket” trap design (**A1**,**A2**)**.** “V” trap design (**B1**,**B2**)**.** Bright-field light microscopy image of “basket” (**A3**) and “V” (**B3**) shape mechanical barriers.

We 3D printed a resin-made chip on a glass slide (Figure 2). This provides convenient access for microscope visualization and can potentially advance other in-vitro assays (e.g., attach and grow cells inside the chip) by adding valuable observational information during the dissolution test. The chip is built from a central compartment with specific barriers designed both for retaining the particles and for evenly dispersing them inside the fluidic device, minimizing their tendency to aggregate. By applying continuous flow instead of using large volume vessels, we were able to achieve sink conditions. The tubes were connected to the chip at both ends by specially designed screwing connectors.

Our design was inspired by Weitz et al., who developed a PDMS microfluidic chip for drug dissolution consisting of mechanical barriers that retain the particles, enabling release testing of infused microparticles [27]. However, while previous reports of chip production used a soft lithography method, we developed a 3D printing protocol. An advantage of a 3D printed chip is its ability to withstand high pressures and flow rates, thus maintaining and ensuring sink conditions, which avoid saturation and mimic physiological conditions.

**Figure 3:**
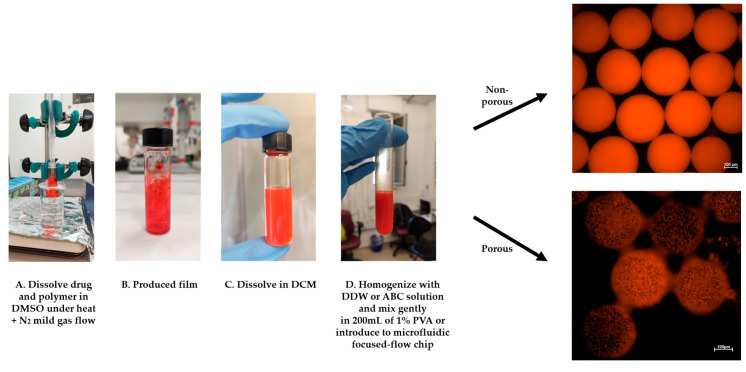
DOX-microsphere (MS) schematic preparation protocol. (**A**,**B**) The solid phase (DOX+PLA) was prepared using a “bain-marie” setup. (**C**) DCM was added and dissolved on the drug-polymer film, (**D**) followed by homogenization with DDW or 1% ammonium bicarbonate (ABC) solution. Finally, the homogenized emulsion was introduced into the microfluidics-focused flow chip platform or instantly poured into 200 mL of 1% PVA to fabricate solidified MS.

In this study, we fabricated porous and non-porous polymeric (PLA) microspheres (MSs) by a solid-in-oil-in-water (S/O/W) protocol (Figure 3). We used a microfluidic focused-flow chip and batch methods for doxorubicin hydrochloride (DOX) encapsulation, which served as a drug model based on a modified oil-in-water-in-oil (O/W/O) protocol.

Our findings indicated that the “V” barrier design exhibits a release profile similar to the control test (using a dialysis sac), as opposed to the “basket” barrier design, which showed a different release profile. Moreover, we studied the effect of the fabrication method and MS morphology on EE, DC and size, along with the effect of pH on the rate and extent of the drug release. We found that MS, fabricated using the microfluidic system, showed better homogeneity and a lower polydispersity index. The drug EE and DC were higher than in the conventional “batch” method. Furthermore, a viability assay conducted on three cell lines demonstrated the safety and the high biocompatibility of the printed material. Altogether, the combination of microfluidics with 3D printing appears to be a powerful platform for designing and producing novel devices for improving preclinical and in-vitro assays. Finally, this study clarifies and accelerates the adoption of greener lab practices by reducing the need for plastic equipment, leading toward more feasible and sustainable research.

## 2. Materials and Methods

### 2.1. Materials

Poly(d,l-lactic acid) (PLA *M*_w_ 75,000–120,000, Sigma-Aldrich, St. Loius, MO, USA), dichloromethane (DCM, Bio-Lab, Jerusalem, Israel), polyvinyl alcohol (PVA *M*_w_ 67,000, Sigma-Aldrich), double distilled water (DDW), ammonium bicarbonate (ABC, NH_4_HCO_3_, Sigma-Aldrich), DOX hydrochloride (E.D.Q.M, Strasbourg, France), isopropanol (Bio-Lab, Jerusalem, Israel) and Freeprint^®^ ortho resin printing polymer (Detax GmbH and Co. KG, Ettlingen, Germany), Coumarin 6 (Sigma-Aldrich, St. Loius, MO, USA).

### 2.2. Microfluidic System

For MS production, the microfluidic system used was from Micronit Microtechnologies (Netherland). The chip (flow-focused design) was made of durable borosilicate glass, its dimensions were 45 mm × 15 mm, with a channel width and depth of 100 and 20 µm, respectively. For the drug release assays, microfluidic dual syringe pumps (Chemyx Fusion 100 Stafford, TX, USA) were used.

### 2.3. 3D Printer and Software

We used a digital light processing—stereolithography printer (Asiga Max-X27 UV, Sydney, Australia). This 3D-printer has a LED light source with 385 nm UV wavelength. The XY pixel resolution of the printer’s projectors was 27 µm and its minimum Z plane resolution was 1 µm. The maximum build size X, Y, Z was 51.8 mm × 29.2 mm × 75 mm, respectively. All the objects were designed with Autodesk AutoCad® software (Q.70.0.0 AutoCAD 2020, San Rafael, CA, USA). The final design model was exported as a stereolithography file and uploaded to Asiga’s software: Asiga composer for 3D printing (v1.1.7, 2020, Sydney, Australia).

### 2.4. Glass Activation

To make transparent prints, we used a glass slide for the surface on which the microfluidic chip was printed. First, we cleaned the glass thoroughly with isopropyl alcohol. The glass slide was activated in 2% of 3-(Trimethoxysilyl)propyl methacrylate (TMSPMA), followed by submersion in 100% ethanol. Finally, we placed the activated glass slide in an oven (set to 105 °C) and allowed it to cool down before starting the printing process.

### 2.5. D Printing Procedure

The printing process was carried out as follows: The build plate was lowered into the vat to a predetermined height and the DLP projected the first slice of the design for a predetermined amount of time. The build plate was raised for a few seconds and lowered again into the vat. The DLP projected the next slices of the design until all layers were printed. Once finished, the printed object was removed from the build plate, rinsed gently with isopropyl alcohol in a sonicator bath (Bandelin) for 3 min, dried using air pressure and finally cured in a UV oven for 5 min (PCU Led, Dreve). The printed chip dimensions were: length (4.5 cm), width (2.5 cm) and height (0.5 cm) for the “basket” and “V” shape design. The dimensions of the interior compartment and the barrier's height are depicted in Figure 1. The inlet flow channel width was 1 mm. When a glass slide was used as the chip substrate, it was first attached to the printer's platform with a few drops of resin polymer. Prior to initiating the printing process, the glass slide height was subtracted from the initial printing point of the platform. At the end of the printing process, the glass with the printed resin was gently detached, cleaned and cured as described above.

### 2.6. DOX Polymeric MS Preparation

DOX-loaded polymeric MS were prepared using a microfluidic flow-focused chip design based on an adjusted S/O/W method [28]. A given amount of polymer was dissolved in DCM and gently poured into a glass vessel containing 5 mg of DOX dissolved in 0.5 mL dimethyl sulfoxide (DMSO). For solvent evaporation, the glass vial was held by tongs and its bottom was dipped inside a warm water (60 °C) bath, and a gentle nitrogen gas stream was applied from above for 5 h. Next, the DOX-polymer film was dissolved in DCM and homogenized (MICCRA homogenizer disperser D-9, Heitersheim, Germany) with 1 mL of pure DDW or 1% ABC solution for 3 min at 8000 rpm for fabrication of non-porous and porous MS, respectively. The porosity was achieved by a gas-foaming technique using ammonium bicarbonate as a gas-foaming agent at the primary emulsion formation (O/W). Then, the homogenized solution was gently perfused into the microfluidic droplet generation chip using a glass syringe, or immediately poured into a 200 mL beaker containing a 1% (*w/v*) aqueous PVA solution. Once the double emulsion formed, small micro gas-bubbles (carbon dioxide and ammonia gas bubbles) spontaneously appear during the solvent evaporation process. The flow-focused chip design consisted of a cross junction, in which the primary homogenized emulsion entered through a central channel and was squeezed at the orifice by two parallel streams of 1% (*w/v*) PVA solution to form a controlled droplet break-up. The fabricated MS (microfluidic and batch synthesis) were stirred in the chemical hood with an overhead propeller at 400 rpm overnight to ensure complete organic solvent evaporation. The MS were washed 3 times with DDW and centrifuged at 5000 rpm for 3 min. Finally, to prepare solidified particles, the MS pellet was resuspended with DDW and frozen overnight at −80 °C and lyophilized (Freezone 6 plus, Labconco, Kansas City, MO, USA) to produce a dry powder of MS for further storage (−20 °C) and characterization.

### 2.7. Recycling Process

The microfluidic chip was easily cleaned at the end of the dissolution assay, making it ready for its next use. First, it was flushed with 100% ethanol for 10 min, followed by a wash with DDW for an additional 10 min at a slow flow rate of 100 µL/min. Finally, the device was dried by applying gentle airflow and then inspected under a light microscope to ensure the removal of all particles and debris.

### 2.8. Morphology and Size Characterization

The morphology of MS was characterized and imaged using a scanning electron microscope (SEM, FEI Quanta 200 microscope, Waltham, MA, USA). A small amount of the samples was spread on a conductive adhesive carbon tape attached to a SEM grid and a thin film of Pd/Au coating sputtered onto the sample (SC7620 Spatter coater, Laughton, East Sussex, UK). The mean MS diameter was measured using Mastersizer 3000 (Malvern, Cambridge, UK).

### 2.9. Encapsulation Efficiency and Drug Content

The drug content (DC) was determined directly by dissolving 5 mg of MS weighed using an analytical scale in 1 mL of DMSO. The solution was filtered and diluted to be detectable within a standard calibration curve range (0.5–20 mg/mL). The absorbance of encapsulated and free DOX in the formulated preparations were measured by a UV–visible spectrophotometer (Biochrom™ Ultrospec 2100 Pro) using a high-precision cell made of quartz SUPRASIL (Hellma™) for DOX absorbance at 480 nm. Prepared MS were weighed after drying. The percentage of EE and the DC of the MS were calculated as follows:

(1) $$\mathrm{EE}\%\;=\;\frac{Mass\;of\;DOX\;in\;MS}{Initial\;mass\;of\;DOX\;}$$

(2) $$\mathrm{DC}\%\;=\;\frac{Mass\;of\;DOX\;in\;MS}{Mass\;of\;MS\;}$$

### 2.10. In Vitro Drug Dissolution Study

DOX polymeric MS (10 mg) were gently injected into a ‘basket’ or a ‘V’ shape microfluidic dissolution chip. The chip was then connected to a syringe filled with PBS release medium (pH = 7.4) containing 0.2% tween 80 and connected to a microfluidic electric pump. As a reference method for the chip drug release assay, we used a dialysis bag (MWCO 1000, Spectra/Por Biotech Regenerated Cellulose, VWR, Atlanta, GA, USA) under constant gentle stirring of 100 rpm. Both systems were maintained at 37 °C for 14 days and samples were collected at predetermined times. Samples of 700 µL were collected from the test tube/vessel solution and immediately measured by UV-vis spectrophotometer at 480 nm wavelength using UV-visible spectrophotometer. Immediately after measuring, the 700 µL were returned to the test tube/vessel. The percentage of release was calculated by normalizing the obtained data at each time point with the cumulative total amount, based on a quantification curve. Finally, to observe the MS morphology at the end of the assay, the MS were gently pumped out of the microfluidic chip, centrifuged, and examined under a SEM microscope. Drug dissolution assays were conducted with MS fabricated with a microfluidic-focused flow chip. Empty particles were used as a control reference (not shown). To validate whether the 3D printed resin absorbs small molecules during dissolution assay, we have conducted an experiment (Supplementary Material Figure S1+S2) comparing absorption of both, hydrophobic (Coumarin 6) and hydrophilic (DOX) molecules in a 3D printed mold as opposed to PDMS mold.

### 2.11. Biocompatibility Assay

H460, A375 and MDA-MB-231 cells (2 × 10^3^ cells/well) were cultured in 10-well Freeprint® (Detax GmbH and Co. KG, Ettlingen, Germany) molds printed on glass slides (Corning, New York, NY, USA). The wells were designed to contain the exact dimensions of wells in 96-well plates, which served as a control. Prior to cell seeding, the printed wells were sterilized under UV light in a laminar-flow hood and coated with Poly-L-Lysine (Sigma-Aldrich). The cells were incubated for 72 h in 37 °C and 5% CO_2_ in their proper conditioned media (RPMI supplemented with FCS 10%, penicillin/streptomycin (P/S) and sodium pyruvate 1% for H460 cells, and DMEM supplemented with FCS 10% and P/S for A375 and MDA-MB-231 cells (Life Technologies, Carlsbad, CA, USA). After incubation, WST-1 reagent (Sigma-Aldrich, St. Louis, MO, USA) was added into each well for viability detection and incubated at 37 °C and 5% CO2 for 1 h. Absorbance was measured at 450 nm using a plate reader (Wallac 1420 VICTOR plate-reader, Perkin-Elmer Life Sciences, Shelton, CT, USA), *n* = 10.

### 2.12. Statistical Methods

In vitro experiments were performed with *n* = 3 unless otherwise stated. All the measurements were statistically analyzed with GraphPad Prism 8.0 (GraphPad Software, Inc., 2019, San Diego, CA, USA) and represented as means ± standard deviations (SDs). To identify statistically significant differences between groups, A Student’s *t*-test was used. A one-way ANOVA followed by Tukey’s test for post-test comparisons were used when more than two groups were compared. Probability values of *p* < 0.05 and *p* < 0.001 were considered statistically significant.

## 3. Results and Discussion

### 3.1. DOX-MS Fabrication Method, EE and DC

The hydrophilic chemotherapy drug DOX was loaded into a hydrophobic polymer core using a S/O/W protocol, either by using microfluidics or in a conventional batch synthesis method. The objective was to compare the effect of the fabrication approach on the encapsulation efficiency, drug content and the polydispersity index. As shown in Table 1, the size of DOX-PMS (DOX-porous MS) and DOX-NPMS (DOX-nonporous MS) varied as a function of the synthesis method. DOX-PMS1 and DOX-NPMS3 fabricated with the microfluidic platform achieved a higher EE (73.8% and 61.5%, respectively) and DC (8.1% and 6.3%, respectively) compared with the DOX-PMS2 and DOX-NPMS4 fabricated by the conventional batch method (56.3% and 44.1%, respectively for EE; and 4.9% and 3.08%, respectively for DC).

Furthermore, DOX-MS1 and DOX-MS3 exhibited lower dispersity indices compared with the DOX-MS2 and DOX-MS4. The microfluidic-based synthesis method revealed higher EE and DC values, with a moderate variation between the tests. This platform enabled exquisite control over fluid flows, and tight control over size and particle morphology [29]. Factors that affect EE and DC included particle size, polymer concentration, drug solubility and uniformity [4,30]. Due to the similarity of fabrication parameters between preparations, we hypothesized that the differences in EE and DC are largely due to the high MS uniformity and the low dispersity index, mainly resulting from the homogenous reaction environment (e.g., laminar flow condition in the microfluidic platform) compared with the conventional batch methods. The latter are often characterized by uneven drug distribution and particle polydispersity due to the applied high-shear stress [31,32]. These results emphasize that microfluidic-based preparations are highly efficient platforms, which offer an optimal method for producing MS, characterized by excellent EE and DC values. Regarding the morphological structure of MS, porous MS in both preparation methods achieved higher EE and DC values. This finding might be explained by the differences in surface area ratio. Porous materials are characterized by open structures, and thus have a high surface area per volume. The absorption process of drugs and other entrapped molecules may be more efficient when subjected to an open network structure that leads to higher drug loading values [33,34]. Hence, to summarize, porous MS has a higher loading capacity potential and, therefore, a greater ability to enhance therapeutic response due to the higher drug concentrations in treated tissues.

### 3.2. In Vitro Drug Release

#### 3.2.1. Mechanical Barrier Shape

**Figure 4:**
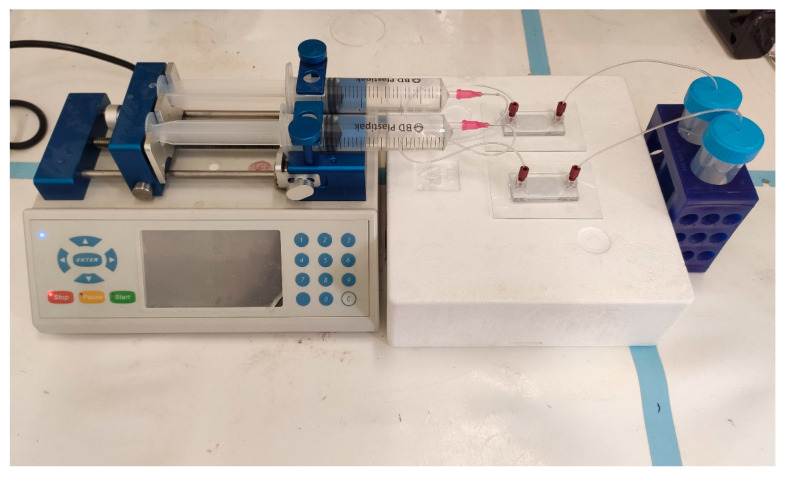
Set-up of the two geometries of 3D printed drug release microfluidic chips connected to the pump. Parallel assays can be performed simultaneously for different chip geometries or for different drug formulations. Once assembled, the system is maintained in 37 °C.

DOX released from a porous MS was fabricated using a microfluidic platform and was evaluated for 14 days in two dissolution devices, that differed in their trap design (“basket” and “V” shapes). These two designs were connected to a microfluidic pump (Figure 4) and were compared to a dialysis sac assay as a standard control method.

**Figure 5:**
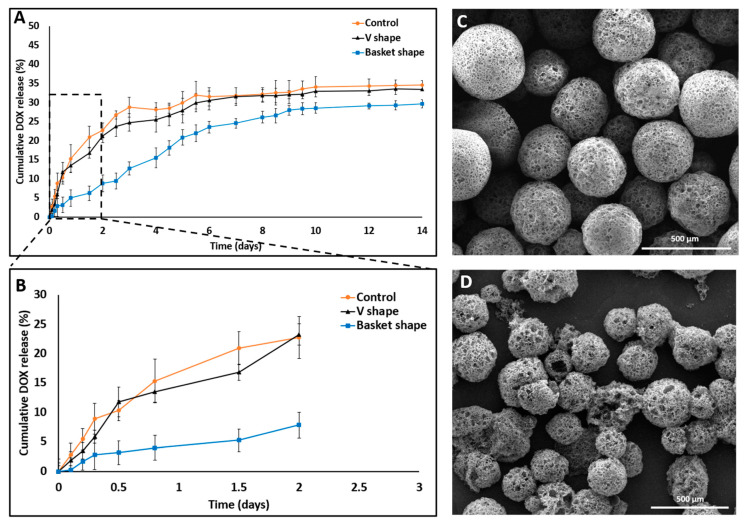
SEM images and DOX release profile studies comparing 3D printed dissolution chips (“V” and “basket” shape) to the dialysis sac method. (**A**) “V” shape chip and dialysis sac methods exhibited similar release behavior both in their release profile and in the total drug released (33%) compared with the “basket” shape chip which exhibited a different release profile and a lower total drug released value (28%). (**B**) Cumulative release in the first 48 h shows a statistically significant difference in the release patterns, with an artificial lower “burst effect” using the “basket” chip. All three experiments achieved a plateau by the end on day 14. (**C**) DOX-PMS1 at day 0. (**D**) DOX-PMS1 at day 14 (empty particles were used as control. *n* = 3; mean ± SD).

All the conditions remained constant and the pH of the released medium was fixed at 7.4. The released amounts of DOX in the “basket”, “V” shape and dialysis method were 28.68% ± 1.15%, 33.4% ± 1.7% and 34.6% ± 2.24%, respectively (Figure 5).

**Figure 6:**
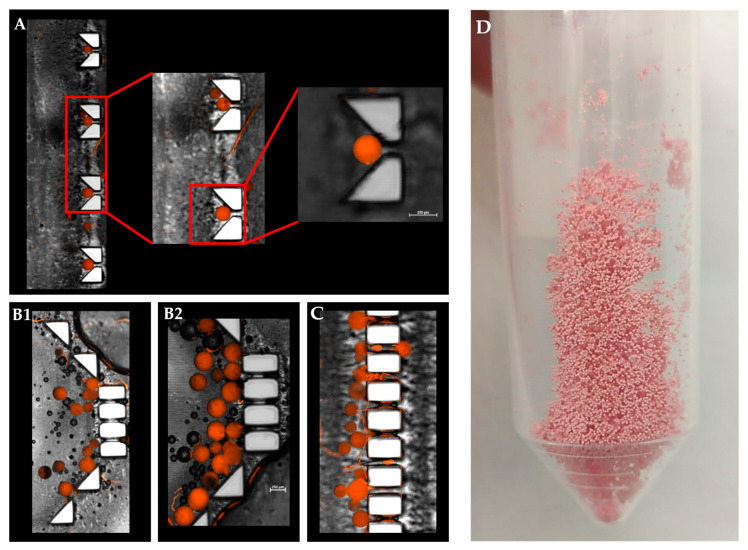
Combined fluorescence and bright-filed microscopy image of a “V” and “basket” shape mechanical barrier. (**A**)**.** Magnified view of a single polymeric microsphere captured in a ‘V’ trap design. (**B1**,**B2**)**.** DOX-PMS captured in the “basket” trap design. (**C**)**.** Trapped DOX-PMS in final pillar barriers before the outlet tube. (**D**) Lyophilized microfluidic based DOX-PMS.

A statistically significant difference was observed between their release profiles within the first 48 h (Figure 5B). Both devices exhibited a sustained drug release profile that reached a plateau after 10 days. Strikingly, although the exact formulation was tested in these two distinct device designs, the pattern of release was different. A careful analysis of the first 48 h revealed that the initial release profiles for both the “V” shape and dialysis bag methods were similar; their DOX release level was 20% or less than that of the “basket” trap structure. These findings might be explained by the accumulation of MS in the single traps introduced into the “V” shape device, thus leading to a broader MS spread over the chip compartment than with the “basket” shape, in which the MS tended to aggregate due to the trap size and design (Figure 6).

Since fluid flows toward lower resistance, this micro-aggregation formed a “cake” that increased the liquid pressure and reduced the available surface area of the MS to fluid flow. This could lead to a lower and slower release rate at the beginning of the release assay [35,36]. Moreover, an initial steeper rise in drug release (“burst effect”) was obtained in the “V” shape trap and the dialysis sac compared with the “basket” trap. This can be attributed to the non-aggregated structure achieved when using a “V” shape dissolution device, which resulted in a higher exposed surface area of the matrix to the flow of the release medium. Therefore, we concluded that the “V” shape design exhibited better and more uniform MS spreading inside the chip, leading to a more reliable method for dissolution assays and thus diminishing the rates for incomplete or misleading drug release data in-vitro. Absorbance of small molecules into PDMS substrate might change the experimental outcomes, and particularly of drug release assay. As shown in Supplemental Material Figure S1+S2, PDMS mold exhibited significant molecules absorption, both hydrophobic and hydrophilic, as opposed to a 3D printed mold. This can result from the fabrication process itself, the vat polymerization via DLP processing of the photopolymer resin following by UV light curing to strengthen the polymer. This emphasizes another advantage of using a 3D printing dissolution chip.

#### 3.2.2. pH Variation

**Figure 7:**
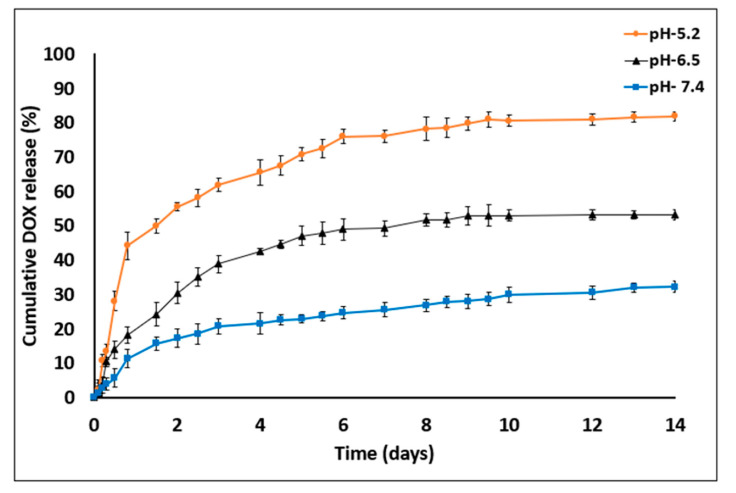
DOX release profile studies in PBS with three different pH value—7.4, 6.5 and 5.2 for 14 days. DOX cumulative release increased in acidic medium conditions (empty particles were used as control. *n* = 3; mean ± SD).

DOX release from porous MS fabricated using a microfluidic platform was assessed at different pH values to test sustainability of the printed chip (“V” barrier design) in various conditions and to predict the in-vivo drug release pattern (Figure 7).

The amounts of DOX released on day 14 were as follows: at pH 7.4, 32.1% ± 1.68%; at pH 6.5, 53.14% ± 1.17% and at pH 5.2, 81.75% ± 1.08%. DOX release was found to be pH dependent, being markedly increased as the pH value decreased. In addition, the device was found to be resistant to certain pH values without any impairment of optical transparency or damage to the MS barriers (the chip was reused over 20 times). In general, sustained DDS formulations have a major role in advanced drug development, including the achievement of higher drug concentrations in tissues and improvement in patient compliance [14,37]. Here, DOX release was evaluated at three pH values that represent various physiological conditions: blood pH (7.2–7.5), tumor microenvironment (6.4–6.8) and endocytic compartments (<5.5). Moreover, DOX was released in a biphasic manner—an initial burst release during the first 48 h followed by a slower release profile. The enhanced drug release at lower pH values might be explained by inherent chemical properties of the DOX molecule. DOX is a weak base containing an amine group that is positively charged (NH3^+^) at acidic pH, making it more soluble in released medium [38,39]. Another possible reason for the burst and increased release is that the acidic environment facilitates polymer degradation via hydrolysis of carboxylic chains and enhances the overall drug release into the medium [25,40].

### 3.3. Resin Biocompatibility

**Figure 8:**
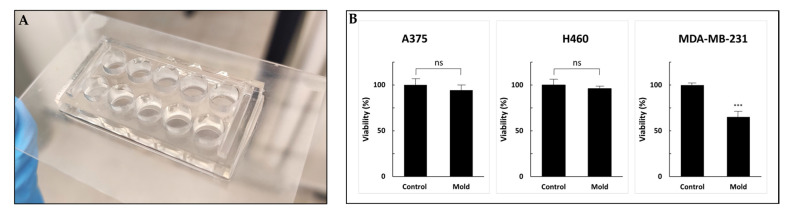
Cell viability (WST-1 cell proliferation assay) to test resin biocompatibility. (**A**) A 96-well design printed on a glass slide. (**B**) A375, H460 and MDA-MB-231 cells were incubated for 72 h followed by the addition of WST-1 reagent. Cell viability was not altered in A375 and H460 cell lines compared with MDA-MB-231 cells that exhibited a statically significant reduction. Values are expressed as means ± standard deviation (SD; *n* = 10). *p* < 0.001 was considered statistically significant and marked with an asterisk. ns: not significant, ***: 0.001.

Although 3D printing is an increasingly common research tool used in the microfluidic realm and in engineering applications, little is known about the cell compatibility and long-term survival of printing materials. Freeprint^®^ resin demonstrated good biocompatibility. Figure 8b shows the viability of three cell lines cultured for 72 h in 10-well molds printed on glass slides (Figure 8).

The control sets were incubated in regular 96-well plates. After three days of incubation, the percentage of viable cells compared to the control (defined as 100%) was 96%, 94% and 65% for A375, H460 and MDA-MB-231, respectively. While A375 and H460 exhibited no alternation in cell growth, the MDA-MB-231 cell line showed 35% inhibition. Although all the printed molds were sterilized and coated with poly-L-lysine to enhance cell adhesion, MDA-MB-231 cells showed statistically significant reduced viability. We hypothesized that further research into possible post-printing treatments, such as using parylene coating, incubation in a cell growth medium that drains secreted toxic chemicals or an alternative method for post-processing with supercritical carbon dioxide, may improve the biocompatibility of printed products and reduce cytotoxicity [41,42,43,44]. Moreover, other viability assays might provide more information regarding the cytotoxic mechanism and other cell functions [45]. Optimal microfluidic photopolymers should exhibit high biocompatibility, and also have high transparency and sufficient resolution to form complex and miniature structures. Overall, the printed device showed good compatibility results and presents a workable basis for future research in the field.

## 4. Conclusions

This work described the feasibility of using an innovative 3D printed dissolution device while combining two revolutionary areas of research: microfluidics and additive manufacturing. This study's objective was to establish a convenient and reliable tool for drug release assays during early research stages and to pave the way for new follow-up studies in these areas. Based on the assays we conducted, DOX presented pH-dependent release, characterized by a burst and enhanced release in an acidic environment. The mechanical barrier shape was found to affect the profile of drug release and its extent. Specifically, the ‘V’ shape dissolution design was found to be more reliable and had a release profile similar to the dialysis method, while the ‘basket’ design presented a different release profile. Furthermore, the 3D printing resin did not exhibit small molecules diffusion and was found to be biocompatible in the cell viability assays. However, further study of its biocompatibility and absorption properties is needed, case-by-case with other cell lines and assays. 

It remains that the technologies described in this study still have a few limitations. The use of a large-scale microfluidic pump together with limited syringe volume required refilling several times during the release assays. A small, pressure-based pump connected to a large-volume vessel might provide an ideal platform for these kinds of studies. Moreover, limitations in resolution precluded the printing of devices that are adjusted to nanoparticles, and the 3D printer stage size limited the ability to print larger chips. Perhaps, in the near future, the advancement of 3D printing technologies and development of new resin materials may lead to higher printing resolutions. This could enable the printing of miniature structures and devices compatible for evaluating nano-particulate DDS.

In conclusion, the ability to print microfluidic chips and other complementary devices using a digital file greatly reduces the need for special equipment and diminishes the need for tedious manufacturing processes (e.g., PDMS-based chips). The use of 3D printing offers low-cost production, characterized by high reproducibility even for non-expert users. Furthermore, these devices are durable and can be recycled multiple times, thus adopting green work habits during pre-clinical research in laboratories. Such technologies are expected to expand beyond pharmaceutical assays and to be used for organ-on-chip applications, and live cell imaging and maintenance.

## Figures and Tables

**Table 1:** Characterization of DOX-loaded MS fabricated via microfluidic and batch methods. Mean diameter, encapsulation efficiency (EE) and drug content are presented as mean ± standard deviation (SD; *n* = 3). DOX-PMS (doxorubicin porous microspheres) DOX-NPMS (doxorubicin non-porous microspheres).

Sample	Method	Mean Diameter (µm)	Encapsulation Efficiency (EE%)	Drug Content (DC%)
DOX-PMS1	Microfluidics	256 ± 8	73.8 ± 0.8	8.13 ± 0.04
DOX-PMS2	Batch	288 ± 56	56.3 ± 0.54	4.95 ± 0.22
DOX-NPMS3	Microfluidics	262 ± 5	61.5 ± 0.3	6.31 ± 0.07
DOX-NPMS4	Batch	273 ± 43	44.1 ± 0.6	3.08 ± 0.47

## Data Availability

Not applicable.

## Supplementary Materials

The following are available online at https://www.mdpi.com/1999-4923/13/1/13/s1, Figure S1: A fast and effective way to fabricate a resin and a PDMS based device by 3D printed mold, Figure S2: Bright-field light and fluorescence microscopy images of PDMS (A) and 3D printed (B) molds of wells containing solutions of 6-coumarin (GPF, green) and doxorubicin (CY3, orange).
