# Supplementary material for: 3D Printed Microfluidic Devices for Drug Release Assays
Source: Pharmaceutics. 2020 Dec 23;13(1):13. doi: 10.3390/pharmaceutics13010013 (PMC7824507; doi:10.3390/pharmaceutics13010013)
Supplement: Supplementary file 1 [file pharmaceutics-13-00013-s001.pdf]

pharmaceutics

MDPI

Supplementary Materials: 3D Printed Microfluidic Devices for Drug Release Assays

Benzion Amoyav, Yoel Goldstein, Eliana Steinberg and Ofra Benny

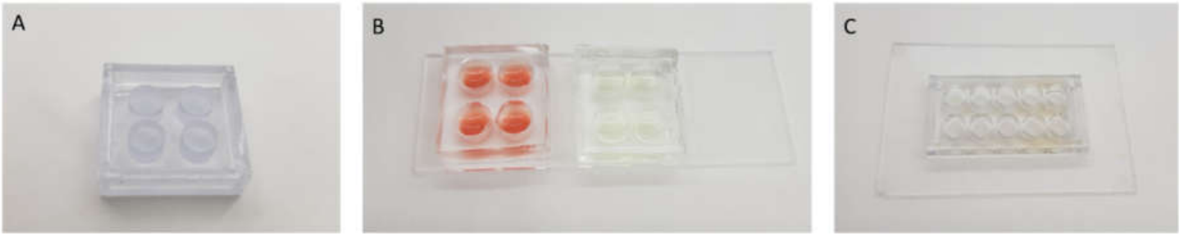

Figure S1. A fast and effective way to fabricate a resin and a PDMS based device by 3D printed mold. **(A)** A 3D printed mold of 96-well dimension printed using a 3D printer. The PDMS resin is poured into the pattern framework and after overnight crosslinking process the PDMS gently peeled from the framework and went through O_2 plasma bonding machine for final glass bonding. **(B)** 150µl of 6-coumarin and doxorubicin solutions placed inside the wells for 24 h incubation in 37 °C. **(C)** Freeprint® molds printed on glass slides. The wells were designed to contain the exact dimensions of wells in 96-well plates.

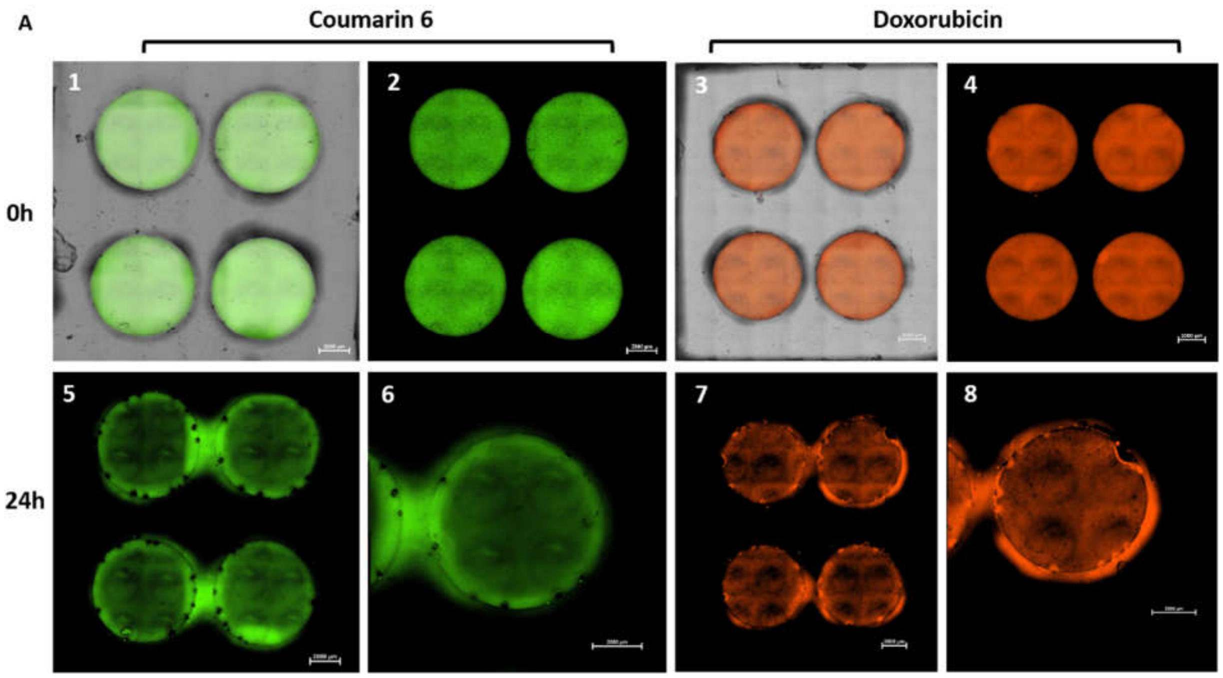

Copyright: © 2020 by the authors. Licensee MDPI, Basel, Switzerland. This article is an open access article distributed under the terms and conditions of the Creative Commons Attribution (CC BY) license (http://creativecommons.org/licenses/by/4.0/).

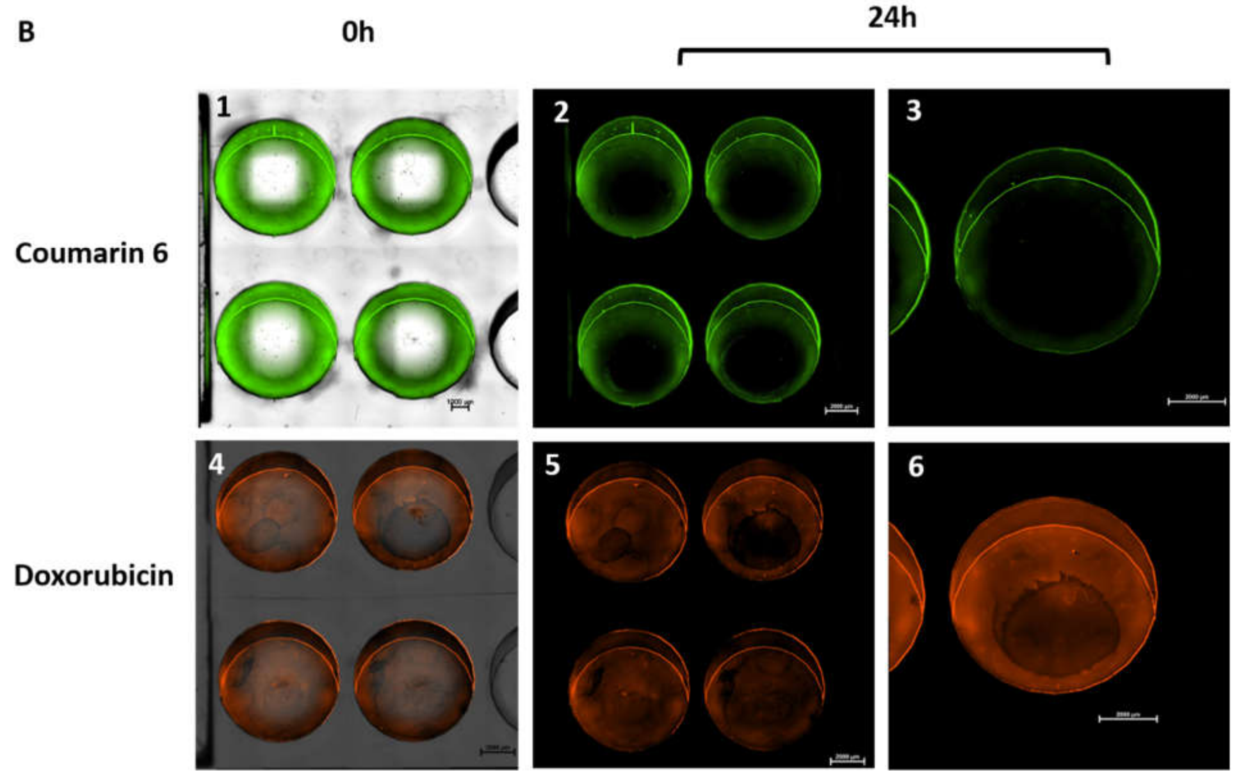

Figure S2. Bright-field light and fluorescence microscopy images of PDMS **(A)** and 3D printed **(B)** molds of wells containing solutions of 6-coumarin (GPF, green) and doxorubicin (CY3, orange). 150µl of 6-coumarin (hydrophobic) and doxorubicin (hydrophilic) solutions placed in 96-well designs of either a PDMS or a 3D printed mold for 24 h incubation in 37 °C. **(A)** Fluorescence images (5,6,7,8) of PDMS mold shows a significant absorption of both molecules to the PDMS substrate. **(B)** Fluorescence images (4,5,6) of 3D printed resin mold shows no absorption of both 6-coumarin and doxorubicin after 24 h. (scale bar— 2000 µm).
